# Perceived evidence use: Measurement and construct validation of managerial evidence use as perceived by subordinates

**DOI:** 10.1371/journal.pone.0266894

**Published:** 2022-04-26

**Authors:** Denise M. Jepsen, Denise M. Rousseau

**Affiliations:** 1 Department of Management, Macquarie Business School, Macquarie University, Sydney, New South Wales, Australia; 2 Heinz School of Public Policy and Management and Tepper School of Management, Carnegie Mellon University, Pittsburgh, Pennsylvania, United State of America; Zhejiang University, CHINA

## Abstract

Despite the promise of evidence-based management as a practice for improving decisions and their outcomes in organizations, little empirical study exists on the effects of evidence use in the workplace. The present research develops a scale to assess subordinate perceptions of managerial evidence use in decision making and provides empirical evidence of the relationships this measure has with established workplace and organizational phenomena. First, scale development studies in four samples, including a field site and MBA courses with students employed full time, show that perceived evidence use can be measured reliably and is distinct from other leadership measures. Second, a cross-sectional study of 308 employees in 18 aged care homes demonstrates a positive relationship between employee perceptions of managerial evidence use and commonly used measures of leader member exchange, trust in supervisor, work-based learning, and organizational performance ratings, and a negative relationship with employee distress. These results suggest implications for leadership and management practices in contemporary, information-rich environments and novel insights into how employees can be affected by managerial evidence use.

## Introduction

Evidence-based management (EBM) is an emerging practice in which managers strive to make conscientious use of the best available evidence in decision-making [1, 2]. The way decisions are made matters. A premise of EBM is that decisions based on the systematic use of evidence are likely to be more acceptable to stakeholders and yield superior outcomes to decisions made in other ways. Conventional decision-making methods tend to be less systematic and more intuitive, typically paying little attention to the quality of the evidence used [3–5].

EBM is part of the broader evidence-based practice (EBP) movement, which promotes the use of the best available evidence from multiple sources. Although practiced in domains as diverse as education, criminal justice, and medicine [6–8], EBP implementation in business management is a relatively recent phenomenon. Progress in promoting EBM is evident by translations of scholarly research for use by managers [9, 10] and a trend toward educating business students in evidence-based practices [1, 11, 12]. However, the consequences of EBM for workers and organizations remain speculative as to date there has been little empirical investigation into the actual effects of managers’ evidence use [13].

There are several reasons why employee perceptions of evidence use by managers might matter. First, perceptions of managerial evidence use can build employee confidence in management, enhancing their good will and advocacy for the organization. Second, perceptions of managerial evidence use can facilitate the teaching role of managers, fostering workplace learning. Third, perceived use of evidence in managerial decisions can support trust-based relationships by reducing employee sense of vulnerability to the effects of decisions based on poor evidence or managerial self-interest, thereby promoting employee well-being. Last, employee ability to discriminate between evidence use in decision making and other management and leadership qualities can provide evidence of the distinct contributions EBM-related practices can make in organizational assessment, development, and research.

This study is the first empirical investigation of the effect that leaders’ evidence use in decisions has on subordinates and subordinate perceptions of the organization. Our research addresses basic questions important at this formative stage in the development of EBM practice. First, are subordinates aware of how their managers use evidence? Second, how do the effects of perceived evidence use in decisions compare with those of established leadership constructs like leader–member exchange (LMX), trust in leadership, and transformational and transactional leadership? Third, what relationships do managerial evidence use have on organizational and employee-related outcomes?

We examined the effects of perceived evidence use (PEU) on subordinate experiences including workplace learning and relationships with their managers. As per Hinkin [14, 15], we developed a measure of PEU and evaluated its psychometric properties. We test the effects of evidence use on conventional and widely used measures of managerial relationships such as LMX and trust and examine its effects on perceptions of organizational performance through intervening effects on workplace learning and relationships with managers.

This research has substantive implications for the literatures of management, leadership, and human resources. First, we develop a reliable and valid measure for employee perceptions of leaders’ evidence use in decision-making to make promote research on managerial evidence use in these domains. Second, we provide evidence of a key mechanism through which perceived evidence use affects employees and organizations, that is, through workplace learning. Third, we find evidence of benefit from perceived evidence use in the form of reduced employee psychological distress. Fourth, we discuss ways employees can have higher regard and advocacy for the quality of their organizations in consequence of PEU.

### Theory and research

Managers and managerial work differ from the practitioners and work conditions characteristic of other EBP domains. Unlike physicians, nurses, psychologists, educators and other professionals, managers can influence their own incentives and often negotiate the performance criteria for which they are responsible [16]. Becoming a manager typically does not require any specific educational credentials; managers, unlike lawyers or physicians, are not bound by professional codes of conduct. Further, managerial performance is often difficult to evaluate because its effects may not be readily observable due to complexity, time lags, or a lack of resources or effort for accurate assessment [17, 18]. The practice of management thus contrasts with other EBP domains where a clearer connection often can be drawn between practitioner decisions and their effects on clients, patients, and other populations [19]. One potential consequence of the unclear connection between managerial decisions and performance may be managerial reliance on intuition, as systematic approaches to decisions may appear less feasible [20]. It is in this context that EBM has emerged as an alternative to conventional managerial practices where limited and nonsystematic evidence use dominates.

### Managerial evidence

The effects of EBP have generally been conceptualized in terms of benefits derived from evidence use in decision-making. These benefits include the use of practices known to be effective and the avoidance of ineffective practices [21]. Evidence use in the EBP context involves conscientious, explicit, and judicious use of the best available evidence [21]. It promotes greater attention to use of multiple sources of evidence in decisions [22, 23].

Several sources of evidence are pertinent to the practice of management [1]. Scientific research is one critical source, where peer-reviewed evidence published in academic journals can be pertinent to managerial decisions. Evidence from scientific research can include both knowledge acquired during a manager’s education as well as research accessed for specific decisions. Managers also use organizational evidence, that is, local, and situational information related to the problem they attempt to address [24, 25]. Organizational evidence is particularly important in EBM because the available scientific evidence is often limited. Local organizational evidence can provide information on situational factors including constraints (e.g., time and resources) and contingencies (e.g., risks), while assessing outcomes for evaluating decision success [24–26]. Organizational evidence plays a critical role when practitioners confront novel circumstances like new technologies or unfamiliar problem situations in which historical evidence is unavailable. In these circumstances, practitioners may need to learn by doing, experimenting with alternative courses of action, and evaluating results [3, 27]. Other sources of evidence available to managers include stakeholder concerns related to problem identification and solution implementation as well as expert judgments from consultants and practitioners [1]. EBM integrates multiple sources of evidence in decision making.

### Subordinate attributions and perceptions of managers

To date, the lack of empirical work on the consequences of evidence use raises critical questions about EBM’s role in organizations [13]. One potential consequence is its impact on employees. Subordinates are a major EBM constituency in their roles as enablers and implementers of managerial decisions. Research suggests that when subordinates become aware of their manager’s evidence use, it can affect how they perceive that manager. Specifically, managers who pay attention to the quality of evidence when making decisions are likely to be viewed by subordinates as fair and trustworthy [28]. Subordinates perceiving that their manager pays attention to evidence implies that these subordinates have access to information regarding the manager’s decisions, particularly how they are made. Thus, leader behavior is a source of support for evidence use [22, 29] and that support itself can promote psychological safety when it comes to employees making their own decisions [30]. Pfeffer and Sutton [4] posit that “when people in the organization see senior executives spending the time and mental energy to unpack the underlying assumptions that form the foundation for some proposed policy, practice or intervention, they absorb a new cultural norm.” We define the perception of evidence use (PEU) to be an employee’s overall impression of their supervisor or manager’s evidence use in making decisions. This definition supposes that supervisors and managers disclose or communicate at least some of their decision process when communicating about a specific decision, change, new way of doing things, or pilot test. This definition focuses on whether evidence use is communicated to the employee. It does not address the specific type or trustworthiness of the evidence used, two additional factors relevant to EBP.

The opinions that subordinates develop regarding their managers are products of their working relationship and interactions, representing interpretations or attributions subordinates make about their manager’s behavior and its effectiveness [31]. Attributions are causal interpretations associated with observed effects or outcomes [32, 33]. Subordinates pay attention to their manager’s behavior and effectiveness across a range of circumstances. In particular, they tend to seek explanations regarding non-routine, surprising, or important decisions [34]. Management decisions with consequences for employees and their organization attract subordinate attention.

Individual employees notice the consistency and distinctiveness of their manager’s behavior when making attributions [35]. These attributions affect not only evaluations of the manager’s performance but also the quality of employee-manager interactions [33]. Such attributions are influenced by the phenomenon of actor-observer bias [36], the tendency to attribute others’ behavior to their internal dispositions and our own behavior to our external environment. As such, subordinates may be more likely to attribute managers’ decisions to the manager’s personal qualities rather than to the decision’s actual circumstances.

With these factors in mind, it follows that what managers communicate regarding their evidence use can influence subordinate attributions regarding the managers’ qualities as people and as leaders. In this study, we tested two attributions subordinates are known to make about their leaders, trust in leaders and the quality of leader-member exchange (LMX).

Trust has been conceptualized as the willingness to be vulnerable to the intentions of another [37]. In subordinate–manager relationships, trust refers to employee beliefs that their manager has positive intentions toward them and behaves in ways that reflect good faith and fair dealing. Managers who communicate the bases for their decisions to subordinates are more likely to be trusted by them than are those who do not [28]. Managers can convey their evidence use by explaining the reasoning behind their decisions. In so doing, they can contribute to both procedural fairness and trust [38]. From the employee perspective, therefore, those who perceive that their managers have disclosed the evidence used to make decisions are more likely to trust those managers. Thus, we hypothesize:

*Hypothesis 1*: PEU is positively related to subordinate trust in the manager.

LMX theory asserts that managers have differentiated relationships with their subordinates based on the quality of interactions a manager has with each subordinate [39]. These differences reflect whether subordinates perceive themselves to be part of an in-group with special access and support by their manager, the basic definition of high LMX. The presence of such a high-quality relationship has been found to reduce employee cynicism [40]. We posit that this relationship with cynicism reflects a link between LMX and the degree to which a manager is perceived to use evidence in making decisions. Employees reporting low LMX tend to believe their organization lacks integrity and makes decisions motivated by political factors (e.g., managerial self-interest), rather than motivated by agreed-upon principles or organizational mission [40]. Conversely, access to inside information regarding managerial decisions may lead subordinates to perceive privileged relationships with their managers, that is, high-LMX relationships. The level of LMX between an employee and his or her manager has been theorized to be influenced by the subordinate’s access to information regarding the manager’s decisions [41]. We extend this argument to hypothesize that LMX is related to subordinate beliefs that the manager uses evidence in making decisions, where evidence use is expected to increase the perception of a quality relationship with the manager. We hypothesize:

*Hypothesis 2*: PEU is positively related to LMX.

### Consequences of evidence use for subordinates and organizations

Managerial evidence use in decision making is likely to impact an organization in multiple ways. In this study we investigate the implications of PEU for employee reports regarding both organizational and individual outcomes. The primary organizational outcome on which we focus is employee evaluation of the overall quality of client care provided by the organisation. The primary individual outcomes we address are employee reports regarding their workplace learning and their psychological well-being (manifest as low employee distress).

#### Workplace learning

Learning is the process of acquiring new or modifying existing knowledge, behavior, skills, values, and practices. Managerial evidence use in decisions is likely to enhance both the managers’ own learning and that of their employees. For example, the quality and degree of informal learning in the workplace can be promoted when managers discuss with subordinates the processes and information they use in decision-making [4]. Workplace learning occurs not only at individual employee level but also at an organizational level through the creation of organization-wide practices that improve performance [42].

Although learning as a general process often occurs in social contexts that permit both observation and direct instruction [43], learning in the workplace is conceptualized to operate across several domains [44] that are affected by managerial evidence use. Two of these domains involve learning through interaction and observation in the workplace. Managers can convey their evidence use through their interactions with subordinates, and their evidence use can be observed in their communications with organizational members, clients, and the public [cf. 45]. The manager’s evidence use can be indirectly manifest in decision making practices demonstrated by coworkers with the same manager [42]. When employees model their decision-making processes on those of their manager, these processes are likely to become normative [4], particularly among those who report directly to the manager [43].

Two additional workplace learning domains involve specific learning processes that managers can model and support. The first, learning by reflecting on task outcomes, involves paying mindful attention to experience, and can be used to both link current experience to past learning and to generate new insights [46]. This learning reflective strategy can be implemented for example, via after-action reviews conducted by managers [1]. The second, experimenting with alternative courses of action, involves the deliberate testing of different ways of solving a problem to compare their outcomes. When confronted by novel or highly uncertain situations in which prior knowledge may not apply, learning by experimenting may be the only evidence-based strategy available [1, 2, 47]. Given the various ways in which managerial evidence use in decisions can promote learning, we hypothesize:

*Hypothesis 3*: PEU is positively related to perceptions of workplace learning.

#### Psychological distress

Workplace demands can place considerable strain on employees. This strain can cause psychological stress [48], a form of negative affect that ranges from anxiety and tension to depression and feelings of hopelessness [49]. Work-related depression reflects a sense of helplessness and frustration regarding employee goals—particularly in terms of achieving important role outcomes. Psychological distress is associated with decreased job satisfaction, reduced job and organizational commitment, lower productivity, and lower work effectiveness [50]. High job demands such as role overload and time pressure are important drivers of employee psychological distress, particularly in the absence of critical support for employees in doing their work [51]. These critical supports include participation in decision-making [50, 51]. Exposure to information that has been used in decision making has been shown to reduce psychological distress [52]; the perceived sufficiency of information has been associated with reduced worry [53] Taken together, it follows that employees whose managers convey their use of evidence in decision making are likely to experience reduced psychological distress. We posit that perceptions of managerial evidence use can mitigate employee psychological distress through the impact that evidence use has on critical mediating factors known to be related to psychological distress. Thus, as posited above, we expect that the positive effect evidence use has on the trust and relationship quality between employees and managers can enhance the emotional support employees experience. Evidence use is also expected to reduce psychological distress on the part of employees through its contributions to workplace learning. This learning prompted by evidence use can materially support employees in dealing with their role demands. Thus, as a function of the relational enhancement and learning associated with evidence use, it is expected to provide both emotional and material support to employees, facilitating their performance and sense of efficacy on the job, thereby reducing psychological distress.

Thus, we hypothesize:

*Hypothesis 4a*: PEU is negatively related to employee psychological distress.
*Hypothesis 4b*: The relationship between PEU and employee psychological distress is mediated by (i) trust in manager (ii) LMX and (iii) workplace learning.

Perceptions of managerial evidence use can also contribute to judgments employees make regarding how well their organization performs. In healthcare, the quality of care provided is a key measure of performance, and evidence use has been found to be predictive of care quality [54]. This effect has been attributed to workplace learning and the making of evidence-based decisions. Employee perceptions of organizational performance are likely to be influenced by local conditions directly experienced by those employees. As their manager’s evidence use is postulated to be a salient aspect of the local work setting, we posit that increased PEU leads employees to also evaluate more positively the quality of care their organization provides. Moreover, through its effect on trust and high-quality workplace relationships, we hypothesize that perceptions of managerial evidence use will indirectly affect how employees rate the quality of care provided by their organization based on the confidence employees have in how organizational decisions are made. Thus, we hypothesize:

*Hypothesis 5a*: PEU has a positive effect on employee assessments of care quality.
*Hypothesis 5b*: The relationship between PEU and employee assessments of care quality is mediated by (i) trust in managers, (ii) LMX, and (iii) workplace learning.

## Methodology

We operationalized PEU in the context of workplace decision-making and implementation of new workplace practices. Consistent with scale-development processes recommended by Hinkin [14, 15], we first developed a set of items conceptualized to reflect PEU. We generated a pool of survey items and tested the psychometric properties of a scale based on these items, examining PEU’s convergent and discrimination validity against LMX, perceived organizational support, organizational justice, and the transformational and transactional dimensions of the Multifactor Leadership Questionnaire [MLQ; 55]. We then evaluated the test-retest reliability of the scale. Finally, we tested the hypothesized relationships between the PEU scale and subordinate assessments of workplace learning, organizational quality of care, and subordinate well-being or distress.

### Phase 1: Development of Perceived Evidence Use (PEU) Scale

#### Study 1: Initial scale development, item reduction and CFA

*Item generation*. To assess the role of evidence use in leadership and workplace perceptions, a measure of managerial evidence use is required. We used a deductive approach to item generation [15] to identify indicators of managerial evidence use that subordinates would be able to observe. Based on a review of the EBM literature, we specified behaviors that could be observed when an individual manager demonstrated attention to relevant evidence when making decisions [2, 4, 56–59]. Focusing on managerial behavior likely to be visible to subordinates, we began with a set of nine items tapping employee perceptions of how their managers made decisions and implemented new ways of working. Items were written as simple, short, single-issue statements with a consistent perspective using language familiar to respondents [14, 15]. The stem was “My manager/s and supervisor/s ….” As recommended by Hinkin [15], responses were based on a five-point Likert scale from 1 (strongly disagree) to 5 (strongly agree).

*Survey administration*. A cross-sectional survey of employees in residential aged care facilities was conducted in regional and metropolitan New South Wales, Australia. The employer was a well-established non-religious charitable institution that managed aged care homes having a mix of self-care, standard care, and high care (dementia) units. There were from 23 to 163 employees per home. Respondents were direct care, catering, facilities maintenance, and support employees.

Most direct care employees in Australia have certificates representing three to six months direct care training and are clinically supervised by registered nurses. Each facility is overseen by a single manager. In larger facilities, the care manager supervises the care and nursing staff, while another supervisor oversees the catering staff. Surveys were distributed to all employees other than the facility manager, with an invitation to participate voluntarily during work hours. Respondents were advised of the university ethics approval and the confidentiality of their responses.

Of 1229 employees invited to participate, 534 (43.4%) responded. Responses with missing data, including those who opened but did not continue the online survey, were excluded, resulting in 308 usable surveys from 18 facilities. Respondent ages ranged from 20 to 77 years, averaging 48 years. Tenure with the organization ranged between 1 and 29 years, with an average of 6.8 years. Demographics of all studies are reported in Table 1.

**Table 1:** Demographics of respondents (all studies).

	Studies 1 and 5		Studies 2 and 4		Study 3	
	(Year 1 aged care) N = 308		(MBA students) N = 121		(Year 2 aged care) N = 367	
	*n*	%	*n*	%	*n*	%
Age, average years	48.0		33.8		46.5	
Tenure, average years	6.8		5.3		5.9	
Sex						
Male	25	8.1	73	60.3	305	83.1
Female	204	66.2	47	38.8	54	14.7
No response	79	25.6	1	0.0	8	2.2
Employment type						
Full-time	79	25.6	101	83.5	74	20.1
Part-time	172	55.8	13	10.7	239	65.1
Casual	19	6.2	5	4.1	50	13.6
No response	38	12.3	2	1.7	4	0.0
Education						
High school	122	40.9			91	15.8
Trade course, cert 3 or 4	82	26.6			193	52.6
University	50	16.2			70	19.1
No response	50	16.2				
Usual roster/shift pattern						
Mostly morning	135	43.8			189	51.5
Mostly afternoon/night	24	7.8			45	12.2
Mixed	84	27.3			98	26.7
No response	65	21.1			35	9.5

*Initial item reduction*. An exploratory principal axis factor analysis [15] with varimax rotation was used to determine the extent to which our PEU items represented one or more factors. Results demonstrated a single factor on which eight of the nine items loaded strongly. Item 1 (“Usually seem to set goals for what they want to achieve”) loaded poorly (.46) and was dropped from further analysis. The eight remaining items demonstrated item-level convergent validity with loadings greater than .8 [60]. Cronbach’s alpha [61] for the eight-item scale was .96, demonstrating good internal consistency. Descriptive statistics, reliabilities, item wording, and factor loadings for all studies are in Table 2.

**Table 2:** Descriptive statistics, reliabilities, and factor analyses of perceived evidence use, 4 and 8 item scales (all studies).

	Study 1	Study 2		Study 3		Study 4				Study 5
	Aged care	MBA students (Time 1)		Aged care (follow-up)		MBA students (Time 2)		MBA students (Time 3)		Aged care
Number of PEU items	9	4	8	4	8	4	8	4	8	4
Number of valid responses to survey items	308	121	121	367	367	90	90	87	87	308
Cronbach’s alpha reliability for PEU scale	.96	.85	.90	.93	.96	.91	.93	.90	.94	.94
Mean	3.70	3.52	3.54	3.80	3.69	3.52	3.53		3.51	3.71
Standard deviation	.85	.93	.88	.88	.85	.90	.86		.86	.87
KMO	.95	.73	.85	.77	.92	.78	.90	.80	.90	.83
Eigenvalue	6.32	2.38	4.36	3.09	5.96	2.84	5.14	2.75	5.17	3.12
Percentage variance explained	70.17	59.5	54.5	77.3	74.5	71.1	64.3	68.77	64.62	79.42
PEU items: “Circle your responses to the following: My manager/s and supervisor/s …:”										
Usually seem to set goals for what they want to achieve.	.46									
1. Make decisions about workplace issues based on evidence.	.80		.73		.83		.82		.84	
**2. Tend to use evidence when implementing a new way of doing things**.	**.86**	**.84**	**.79**	**.85**	**.86**	**.78**	**.81**	**.86**	**.84**	**.85**
**3. Tell me about the evidence for implementing a new way of doing things**.	**.90**	**.80**	**.79**	**.90**	**.88**	**.90**	**.87**	**.89**	**.90**	**.90**
4. Ask me for feedback for my opinion after implementing a new way of doing things.	.89		.71		.87		.68		.74	
5. Involve me in research on workplace issues.	.83		.69		.82		.73		.75	
**6. Give me/us the information on the success (or otherwise) of a trial or a new way of working**.	**.91**	**.67**	**.72**	**.89**	**.89**					**.91**
**7. Like to evaluate the success of a new way of working**.	**.91**	**.76**	**.77**	**.88**	**.90**					**.91**
8. Share their experiences of workplace trials, changes, and new implementations with other supervisors and managers.	.88		.71		.86					

*Confirmatory factor analysis*. To further assess construct validity, we conducted confirmatory factor analyses [CFA; 62]. The first CFA specified one factor with eight items corresponding to the results of our exploratory analysis. A second two-factor CFA resulted in a high correlation (.92) between the two factors. The fit statistics for the two-factor model were satisfactory when compared with a one-factor model, as shown in Table 3. The first factor, consisting of items 2, 3 and 4, was labeled *evidence use*. The second factor, consisting of items 6, 7, 8 and 9, was named *evidence sharing*. Loadings on all items were from .83 to .94. Cronbach’s alphas for the separate scales were .93 and .95 respectively. After the low-loading item 5 was removed, a third, seven-item, two-factor model demonstrated poor discrimination between the factors (*r* = .92). As the scale properties did not improve with the two poorly discriminating factors, we retained the more parsimonious one-factor model.

**Table 3:** Fit statistics for competing models for perceived evidence use (Study 1).

Model	*x* ^2^	Df	*x*^2^/Df	TLI	CFI	RMSEA	SRMR
PEU, 8 items, single factor	190.22	20	9.51	.91	.93	.17	.03
PEU, 8 items, two factors	92.88	19	4.89	.96	.97	.11	.02
PEU, 7 items, two factors	65.22	13	5.02	.96	.98	.11	.02

*Note*. PEU = perceived evidence use; TLI = Tucker Lewis index; CFI = comparative fit index; RMSEA = root mean square error of approximation; SRMR = standardized root mean square residual.

#### Study 2: Convergent and discriminant validity with leader–member exchange, perceived organizational support, and organizational justice

As there are a plethora of constructs concerning employee perceptions of their managers, it was important to test whether PEU is distinct and then to establish the nomological network of relationships PEU has with existing measures (Hinkin, 1998). To evaluate the discriminant validity of PEU, we examined its potential overlap with three widely used potentially relevant measures. First, we examined the relationship between PEU and LMX. LMX reflects the quality of the dyadic relationship between a manager and their subordinates [39], a primary indicator of mutual support and quality of exchange. Second, we examined the link between PEU and perceived organizational support (POS), the latter reflecting the quality of the relationship individuals experience with their employers [63]. Finally, because perception of evidence use may be construed as an aspect of organizational fairness or justice, we examined the relationship between PEU and the four forms of organizational justice: procedural, distributive, interpersonal, and informational justice [64].

Respondents in this study were 121 part-time students in three MBA human resources classes in an Australian business school, aged from 23 to 57 years (average 33.8) and 60.3% male, as shown in Table 1. The majority (83.5%) were employed full-time, with 10.7% part-time, 4.1% casual, and 1.7% unspecified. Respondents reported one to 20 years tenure with their employers (average 5.3), and one to 20 years with their current managers (average 2.7). Respondents were informed of the voluntary and confidential nature of the study and the university’s ethics committee approval. No compensation or class credit was given for participation. Paper surveys were distributed in class and participants were given ten minutes to complete them. Those who chose to participate returned their completed survey to a large envelope, which was then sealed and handed to the researcher.

*Measures*. *Perceived evidence use*. Two versions of the PEU scale were created for Study 2. The first version (PEU8) comprised the eight items retained from Study 1. The second was a more parsimonious four-item PEU scale (PEU4). PEU4 used the four items with the highest loadings: “tend to use evidence when implementing a new way of doing things,” “tell me about the evidence for implementing a new way of doing things,” “give me/us the information on the success (or otherwise) of a trial or a new way of working,” and “like to evaluate the success of a new way of working.” The stem for both was “Regarding your manager/s and supervisor/s, he, she or they…” Cronbach’s alphas for the eight- and four-item scales were .90 and .85 respectively.

*Leader–member exchange*. LMX refers to the perceived quality of respondents’ relationships with their supervisors, including the extent to which their supervisor understands their needs and takes time to support them. An example from the seven-item scale [39] is “how well does your supervisor understand your job problems and needs?” Cronbach’s alpha was .90.

*Perceived organizational support*. POS refers to respondent perceptions of the level of support provided by management, including the extent to which management cares about staff well-being and the amount of help available. A nine-item scale was used with seven-point responses from “disagree strongly” to “agree strongly” [63]. A sample item was “management cares about my opinions.” Cronbach’s alpha was .70.

*Perceived organizational justice*. Twenty items were used to measure respondents’ perceptions of organizational justice, with seven procedural justice items, four distributive justice items, five interpersonal items, and four informational justice items [64]. The stem of the procedural and distributive justice items was “fairness of the procedures used for your pay and promotions: To what extent ….” One procedural justice example was “have you had influence over the results arrived at by those procedures?” A distributive justice example was “do the benefits reflect the effort you have put into your work?” The interpersonal and informational scales used the stem, “about your business unit manager.” An interpersonal justice example was “have they treated you in a polite manner?” An informational justice example was “have they explained the procedures thoroughly?” A five-point Likert scale, from 1 (not at all) to 5 (to a great extent) was used. Cronbach’s alphas were .89, .95, .93, and .93, respectively.

Descriptive statistics, reliabilities and scale-level correlations for this study are reported in Table 4. All correlations were in the expected direction. We note the moderate correlations of PEU8 with procedural, distributive, interpersonal, and informational justices (.49, .50, .42, and .52 respectively) and with POS (.37). Table 4 also shows comparable correlations of PEU8 and PEU4 with all study variables.

**Table 4:** Descriptive statistics, correlations, discriminant validity reliabilities of PEU, LMX, POS, OJ (Studies 2 and 4).

	Mean	SD	1	2	3	4	5	6	7	8	9	10	11	12
1. PEU4, Time 1	3.52	0.93	(.85)											
2. PEU4, Time 2	3.52	0.90	.74**	(.91)										
3. PEU4, Time 3	3.57	0.90	.71**	.88**	(.90)									
4. PEU8, Time 1	3.52	0.88	.95**	.75**	.74**	(.90)								
5. PEU8, Time 2	3.53	0.86	.73**	.97**	.90**	.76**	(.93)							
6. PEU8, Time 3	3.51	0.86	.69**	.89**	.97**	.74**	.93**	(.94)						
7. LMX	3.83	0.63	.13	.27	.41*	.30	.33	.45*	(.90)					
8. POS	4.60	0.65	.24	.41*	.49*	.37*	.51**	.55**	.69**	(.70)				
9. OJ Procedural	3.12	0.93	.46**	.48**	.48**	.50**	.52**	.52**	.42*	.49**	(.89)			
10. OJ Distributive	3.23	1.18	.49**	.40**	.41**	.51**	.49**	.46**	0.22	.36*	.76**	(.95)		
11. OJ Interpersonal	4.14	0.90	.37**	.44**	.45**	.42**	.47**	.47**	.49**	.55**	.45**	.43**	(.93)	
12. OJ Informational	3.33	1.08	.47**	.58**	.57**	.52**	.62**	.61**	.48**	.55**	.60**	.58**	.60**	(.93)

*Note*. Cronbach’s alpha reliabilities are in brackets on the diagonal. PEU = perceived evidence use; LMX = leader–member exchange; POS = perceived organizational support; OJ = organizational justice.

To further assess the validity of the PEU construct, we used a CFA to test its discriminant validity with LMX, POS, and the four forms of justice. For LMX, we tested one model with LMX and PEU combined into a single factor, and another where the constructs were modeled on two separate factors. Only the separated constructs, the two-factor model, demonstrated an acceptable fit. For POS, we tested one model with POS and PEU combined into a single factor, and another where the constructs were modeled on two separate factors. Again, only the separate constructs in the two-factor model demonstrated an acceptable fit. For organizational justice, we tested one model with all four justice factors and PEU combined into a single factor, another with three justice factors and PEU combined with the procedural justice factor, and a third model with all five factors separated. Only the separate constructs in the five-factor model demonstrated an acceptable fit.

Results demonstrate PEU has good discrimination from LMX, POS, and organizational justice. Because there is no single test of fit [65], Table 5 reports the chi-square test statistic, the root mean square error of approximation (RMSEA) in which values greater than .10 indicate a poor fit, the standardized root mean square residual (SRMR) in which values less than .08 indicate a good fit, and the comparative fit index (CFI) in which values greater than .90 indicate an acceptable fit [66–69]. Results are given for both the four-item and eight-item versions of the scale.

**Table 5:** Fit statistics for discriminant validity LMX, POS, OJ and MLQ (Studies 2 and 3).

Model	Dataset	x^2^	Df	*x*^2^/Df	TLI	CFI	RMSEA	SRMR
Study 2: PEU and LMX leadership								
A: PEU8 two factors	MBA *n* = 121	181.95	89	2.04	0.90	0.91	0.09	0.07
B: PEU8 one factor (all items)		389.11	90	4.32	0.67	0.72	0.17	0.11
A: PEU4 two factors		85.97	43	2.00	0.92	0.94	0.09	0.06
B: PEU4 one factor (all items)		220.41	44	5.01	0.70	0.76	0.18	0.12
Study 2: PEU and perceived organizational support								
A: PEU8 two factors	MBA *n* = 121	245.91	118	2.08	0.89	0.91	0.10	0.07
B: PEU8 one factor (all items)		495.12	120	4.13	0.69	0.73	0.16	0.11
A: PEU4 two factors		131.26	64	2.05	0.92	0.94	0.09	0.05
B: PEU4 one factor (all items)		311.43	66	4.72	0.72	0.77	0.18	0.11
Study 2: PEU and four organizational justice factors								
A: PEU8 five factors	MBA *n* = 121	565.94	340	1.67	0.91	0.92	0.07	0.07
B: Four factors: PEU8		838.78	344	2.44	0.81	0.82	0.11	0.09
C: PEU8 one factor (all items)		1626.31	350	4.65	0.51	0.55	0.17	0.12
A: PEU4 Five factors		398.37	242	1.65	0.93	0.94	0.07	0.07
B: Four factors: PEU4		543.55	246	2.21	0.87	0.88	0.10	0.08
C: PEU4 One factor (all items)		1327.14	252	5.27	0.52	0.57	0.19	0.12
Study 3: PEU8 and MLQ 5 transformational leadership factors								
A: Six factors: PEU8 and 5 transformational factors	Year 2 *n* = 388	1310.57	335	3.91	0.92	0.93	0.09	0.03
B: Five factors: PEU8 (loaded on intellectual stimulation factor)		2430.96	341	7.13	0.82	0.84	0.13	0.08
C: One factor all items		3651.62	350	10.43	0.73	0.75	0.16	0.08
A: Six factors: PEU4 and 5 transformational factors		881.39	237	3.72	0.93	0.94	0.09	0.03
B: Five factors: PEU4 (loaded on intellectual stimulation factor)		1519.52	243	6.25	0.86	0.88	0.12	0.05
C: PEU4 One factor (all items)		2189.54	252	8.69	0.79	0.81	0.15	0.06
Study 3: PEU8 and MLQ 4 transactional leadership factors								
A: Five factors: PEU8 and 4 transactional factors	Year 2 *n* = 367	883.24	242	3.65	0.89	0.91	0.09	0.06
B: Four factors: PEU8 (loaded on mgmt. by exception passive factor)		1587.07	246	6.45	0.78	0.81	0.12	0.11
C: PEU8 One factor (all items)		2844.41	252	11.29	0.59	0.62	0.17	0.14
A: Five factors: PEU4 and 4 transactional factors		524.55	160	3.28	0.91	0.93	0.08	0.06
B: Four factors: PEU4 (loaded on mgmt. by exception passive factor)		1181.13	164	7.20	0.76	0.79	0.13	0.11
C: PEU4 One factor (all items)		2343.00	170	13.78	0.51	0.56	0.19	0.15

*Note*. PEU = perceived evidence use; MLQ = Multifactor Leadership Questionnaire; TLI = Tucker Lewis index; CFI = comparative fit index; RMSEA = root mean square error of approximation; SRMR = standardized root mean square residual; mgmt = management.

#### Study 3: Convergent and discriminant validity with multifactor leadership questionnaire

We further tested the discriminant validity of PEU to establish its differentiation from the MLQ, a well-established measure of leadership. We collected data in a follow-up survey of employees in the same aged care organization as Study 1 one year later. This survey was distributed on paper and online over a two-month period. There were 603 responses out of 1413 employees, a 42.6% response rate. After removing outliers and responses with missing data, including those who opened but did not continue the online survey, there were 367 usable responses. Respondents were mostly female (83.1%), aged 17 to 79 years (average 46.5), and had a tenure of zero to 30 years (average 5.9).

*Leadership measure*. The 36-item MLQ [55] was used. The MLQ measures a broad range of leadership types from passive leaders to leaders who give contingent rewards to followers, and leaders who transform their followers into leaders themselves. The five transformational leadership scales of inspirational motivation, idealized influence (attitudes), idealized influence (behaviors), intellectual stimulation, and individual consideration each have four items (e.g., “articulates a compelling vision of the future”, “acts in ways that builds my respect”). Cronbach alphas for these scales were all above .86.

The four transactional leadership scales of contingent reward, management by exception (active), management by exception (passive), and laissez-faire each have four items (e.g., “discusses in specific terms who is responsible for achieving performance targets,” “demonstrates that problems must become chronic before taking action”). The stem was “think about your supervisor or manager, the person who usually controls your work or to whom you report. Please judge how frequently each statement fits the person you are describing. The person I am rating…” The response scale was from 0 (not at all) to 4 (frequently, if not always). Cronbach alphas were all above .78.

Descriptive statistics, correlations and Cronbach alphas for this study are reported in Table 6. Correlations were all in expected directions. Correlations between the PEU and MLQ transformational leadership scales were between .67 and .72, which we note is less than the correlations between the five of MLQ transformational scales (all five of which ranged from .75 to .89). Correlations between PEU and MLQ transaction scales were similar for contingent reward, non-significant for active management by exception, and moderately negative for passive management by exception and laissez-faire.

**Table 6:** Descriptive statistics, correlations, reliabilities for discriminant validity of PEU and MLQ (Study 3).

	Mean	SD	1	2	3	4	5	6	7	8	9	10	11
1. PEU4	3.79	0.88	(.93)										
2. PEU8	3.68	0.85	.98**	(.96)									
MLQ transformational scales													
3. Idealized influence attributed	3.82	1.00	.67**	.70**	(.89)								
4. Idealized influence behavior	3.82	1.01	.68**	.70**	.88**	(.92)							
5. Inspirational motivation	3.75	1.09	.68**	.68**	.84**	.89**	(.95)						
6. Intellectual stimulation	3.67	1.09	.70**	.73**	.82**	.84**	.84**	(.95)					
7. Individual consideration	3.59	1.05	.67**	.69**	.84**	.77**	.75**	.76**	(.86)				
MLQ transactional scales													
8. Contingent reward	3.62	1.07	.68**	.69**	.71**	.71**	.70**	.69**	.70**	(.90)			
9. Mgmt by exception active	2.96	1.09	.09	.15**	.11*	.12*	.16**	.17**	.15**	.22**	(.78)		
10. Mgmt by exception passive	1.88	0.98	-.58**	-.49**	-.51**	-.51**	-.48**	-.47**	-.44**	-.40**	.13*	(.80)	
11. Laissez-faire leadership	1.82	0.99	-.46**	-.46**	-.48**	-.50**	-.45**	-.44**	-.43**	-.41**	0.08	.73**	(.84)

*Note*. Cronbach’s alpha reliabilities are in brackets on the diagonal. PEU = Perceived evidence use; MLQ = Multifactor Leadership Questionnaire; mgmt = management.

CFAs were conducted to establish discriminant validity between PEU and each of the MLQ measures. First, we examined the transformational MLQ scales. To assess discrimination against the five-factor transformational leadership model, we tested one-, five-, and six-factor models. Only the six-factor model had an acceptable fit, indicating that PEU is a separate construct, distinct from each of the five MLQ transformational factors.

To assess discrimination against the four-factor transactional leadership model, we tested one-, four-, and five-factor models. Again, results show only the five-factor model (PEU combined with intellectual stimulation factor) had an acceptable fit, indicating that PEU is a separate discrete construct from the four MLQ transactional factors. Table 5 includes goodness-of-fit indices for both the four- and eight-item PEU scales. Notably, PEU4 and PEU8 performed similarly.

#### Study 4: Test-retest reliability

Although some managers might use evidence on single occasions when responding to significant events, our expectation was that subordinates would have a general perception of how their managers tend to use evidence. Thus, to test the stability of the four- and eight-item PEU measures, we assessed test-retest reliability.

Respondents were the same MBA students who participated in Study 2. One week after the initial survey, a second survey with all eight PEU items was distributed. A week later, a third survey with the same items was distributed. Respondents were asked to give their student ID numbers on each occasion to link the three surveys. Although 148 responses were received, 27 respondents did not complete at least two of the three surveys. After removing incomplete responses, there were 121 usable responses at Time 1 (Study 2), 91 at Time 2, and 87 at Time 3.

Descriptive statistics, correlations, and Cronbach alpha reliabilities are shown in Table 4. The PEU8 showed high correlations between Time 1 and Time 2 (*r* = .76), Time 1 and Time 3 (.73), and Time 2 and Time 3 (.93). PEU4 showed similarly high correlations between Time 1 and Time 2 (*r* = .74), Time 1 and Time 3 (.71), and Time 2 and Time 3 (.84). These results indicate good test-retest reliability for both PEU4 and PEU8. Each version correlated highly at across administrations (from .93 to .97), suggesting the longer version of the scale added little value. Table 7 shows the CFA fit statistics for each version of the PEU scale at Times 1, 2, and 3.

**Table 7:** Fit statistics for reliability study for PEU 4 and 8 item scales (Study 4).

	N	*x* ^2^	Df	*x*^2^/Df	TLI	CFI	RMSEA	SRMR
PEU 4 items (Time 1)	121	22.12	2	11.06	.74	.91	.29	0.07
PEU 4 items (Time 2)	91	15.47	2	7.74	.79	.93	.27	0.06
PEU 4 items (Time 3)	87	15.87	2	7.93	.77	.92	.28	0.07
PEU 8 items (Time 1)	121	87.13	20	4.36	.83	.88	.17	0.06
PEU 8 items (Time 2)	91	65.93	20	3.30	.86	.90	.16	0.06
PEU 8 items (Time 3)	87	70.07	20	3.50	.84	.88	.17	0.06

*Note*. PEU = perceived evidence use; TLI = Tucker Lewis index; CFI = comparative fit index; RMSEA = root mean square error of approximation; SRMR = standardized root mean square residual.

Together, these analyses demonstrate the PEU scale as robust, reliable, and appropriately discriminant from standard measures of LMX, POS, organizational justice and leadership.

### Phase 2: Hypothesis testing, Study 5

We now address the effects that perceptions of managerial evidence use can have on subordinates, perceptions of their manager, and potential consequences for themselves and their organization. Hypothesis testing was conducted using data obtained from a survey administered to employees in the residential aged care organization described in studies 1 and 3.

#### Measures

Responses to survey items were based on five-point Likert scales (i.e., 1 = strongly disagree to 5 = strongly agree) unless otherwise indicated. Items were averaged to form scale scores, where higher scores indicated a greater degree of the construct.

*Perceived evidence use*. Given the equivalence of the four- and eight-item versions of the scales (study 4), we used the more parsimonious four item PEU4 scale outlined above. Reflecting on the dimensions of evidence use Rousseau [5] describes, the stem was “My manager/s and supervisor/s.” Cronbach’s alpha was .94.

*Leader–member exchange*. We used the seven-item LMX measure [39] described in Study 2. Cronbach’s alpha was .94.

*Trust in supervisor*. Five items [70] were used to assess trust in supervisors. Examples of these items included “my supervisor would never try to gain an advantage by deceiving workers” and “I have complete faith in the integrity of my supervisor.” Cronbach’s alpha was .78.

*Work-based learning*. 12 items of a multi-dimensional scale were used to assess employee perceptions of learning at work [44]. This model specified four dimensions of work-based learning (WBL) that reflected learning potential in the workplace, with three items each for the dimensions of reflection, experimentation, colleagues and supervisor. The stem was “Please indicate to what degree these statements are applicable to your current work situation.” Sample items included “in my work I am given the opportunity to contemplate about different work methods” (reflection), “in my job I can try different work methods even if that does not deliver any useful results” (experimentation), “my colleagues tell me if I make mistakes in my work” (colleagues), and “my supervisor helps me see my mistakes as a learning experience” (supervisor). Responses were from 1 (not applicable at all) to 5 (completely applicable). Two items (“In my job I can try different work methods even if that does not deliver any useful results” and “my colleagues are eager to collaborate with me in finding a solution to a work problem”) were removed due to poor CFA loadings. Cronbach’s alphas for these scales were .89, .81, .73, and .88 respectively.

*Quality of care*. Two items assessed respondents’ perceptions of the quality of care their facility provided. These were “I would recommend this facility to my family and friends” and “if I had to go into a facility one day in the future, I think I would be happy to be at this facility.” These items were loaded on a single factor with a Cronbach’s alpha of .91.

*Psychological distress*. A modified Kessler Psychological Distress Scale [49], a global measure of distress, was used to assess employee depression and anxiety over the preceding four weeks. The stem for all items was “Please indicate, in the past four (4) weeks, about how often did you feel…” The items included “hopeless” and “that everything was an effort.” CFA revealed low loadings for five items (“Tired out for no good reason,” “nervous,” “so nervous that nothing could calm you down,” “restless or fidgety,” and “so restless you could not sit still”), which were removed. Cronbach’s alpha for the remaining five items was .85.

*Control variables*. Consistent with established practice in leadership research [71, 72], we controlled for respondent demographics. Because human capital theory [73] postulates that individual characteristics such as tenure and education affect employee attitudes and behavior, we controlled for age, organizational tenure, and education, the latter using dummy variables (0 = high school or certificate; 1 = undergraduate degree or higher). We also used dummy variables to control for sex (0 = female; 1 = male) and, given the possible impact of lesser or greater opportunities for observing managerial evidence use, shift (0 = day or mixed shift; 1 = night shift).

*Analysis*. We recognized the potential for a nesting effect, given respondents were employed at facilities in 18 different locations. We tested for such a nested facility effect at the individual level to determine if multilevel modeling were warranted. Using SPSS (Version 22), regressions were conducted on each of the dependent variables to test for facility-level differences. First, dummy codes were created for each facility (1 = facility number; 0 = not that facility). For example, Fac01 was coded as a dummy variable for all respondents so that if the respondent was from Facility 1, then Fac01 was 1. If the respondent was from another facility, then Fac01 was coded as 0. This was repeated for 14 facilities, including Fac98 for respondents from facilities with fewer than five respondents, and Fac99 for the 31 respondents who did not give their facility code. Regression results indicated that only respondents from Facility 2 differed and only in terms of the outcomes psychological distress and perceived quality of care. As such, we judged a general nesting effect to be unlikely, making multilevel analysis inappropriate and thus hypotheses were tested using individual-level analyses.

## Results

Means, standard deviations, correlations and Cronbach alpha reliabilities for this data are shown in Table 8. All correlations were in the expected directions. PEU4 correlated highly with LMX (*r* = .73), trust in supervisor (.67), WBL supervisor (.60), and moderately with WBL reflection (.43) and WBL experimentation (.40), but not with WBL colleagues (.11). PEU4 also correlated negatively with psychological distress (-.33) and positively with quality of care (.60). Similarly, LMX correlated negatively with psychological distress (-.34) and positively with quality of care (.64). Trust in supervisor followed the same pattern, correlating negatively with psychological distress (-.35) and positively with quality of care (.59). Psychological distress correlated negatively with quality of care (-.38).

**Table 8:** Descriptive statistics, correlations, reliabilities for hypothesized model (Study 5).

	Mean	SD	1	2	3	4	5	6	7	8	9	10
1. PEU4	3.71	0.86	(.94)									
2. PEU8	3.69	0.85	.98**	(.96)								
3. LMX	3.66	0.98	.73**	.76**	(.94)							
4. Trust in supervisor	3.87	0.78	.59**	.61**	.78**	(.78)						
5. WBL reflection	3.77	0.96	.43**	.44**	.40**	.36**	(.89)					
6. WBL experiment	3.21	1.00	.40**	.41**	.36**	.32**	.72**	(.81)				
7. WBL colleagues	3.80	0.92	0.11	.12*	.13*	.11**	.26**	.28**	(.73)			
8. WBL supervisor	3.77	1.08	.60**	.61**	.65**	.56**	.47**	.42**	.36**	(.88)		
9. Psychological distress	1.35	0.59	-.33**	-.34**	-.34**	-.34**	-.27**	-.22**	-0.03	-.33**	(.85)	
10. Quality of care	3.95	1.01	.60**	.62**	.63**	.53**	.36**	.34**	.20**	.51**	-.38**	(.91)

*Note*. Cronbach’s alpha reliabilities are in brackets on the diagonal. PEU = perceived evidence use; WBL = work-based learning.

*Correlation is significant at the 0.05 level (2-tailed).

**Correlation is significant at the 0.01 level (2-tailed).

### CFAs to test discriminant validity

A series of CFAs verified the discrimination among path model variables. A CFA was conducted to assess discriminant validity between PEU and the four WBL scales. The WBL colleagues scale demonstrated low correlations with other variables (e.g., .02 with PEU) and was dropped from further analysis.

As the data were obtained through same-respondent self-report surveys, we took precautions to reduce the risk that common method variance could lead to biased results. With respect to communication with respondents, we detailed how respondent data would be kept confidential, reassured them that neither their supervisors nor any person in their organization would see their responses and reassured that there were no right or wrong answers. We detailed the university ethics approval and processes for collecting the data. With respect to survey preparation, we used established scales and items in addition to the PEU measure we developed [74]. With respect to post hoc statistical analyses, we used SPSS to estimate a one-factor model on which all items were loaded. We conducted Harman’s test for common method variance with an exploratory factor analysis of all variables. Ultimately, and consistent with latest research demonstrating a very low probability of distortion from common method variance [75], these results suggest little likelihood of common methods bias [72].

We used structural equation modeling to analyze the strength, direction, and significance of relationships between variables using AMOS [Version 25; 62]. Assumptions relating to the ratio of sample size to variables, skewness, and kurtosis were satisfied [69].

### Hypothesis testing

We examined the structural path for the hypothesized model, with the PEU4 independent variable mediated by LMX, trust, and each of the four WBL variables (reflection, experimentation, colleagues, and supervisors). After removing non-significant hypothesized paths, resulting fit statistics were well within the acceptable range [χ^2^ (9, *N* = 308) = 25.026, *p* < .001; RMSEA = .076; SRMR = .0440; CFI = .984].

Hypotheses 1 and 2 predicted PEU would be positively related to employee perceptions of both trust in their manager and LMX. The paths from PEU4 to both LMX (.34) and trust in supervisor (.40) were significant (*p* < .01), supporting both hypotheses.

Hypothesis 3 predicted PEU would be positively related to perceptions of WBL. While the regression path from PEU4 to WBL supervisor was strong (.60) and the path to WBL reflection (.23) both were significant (*p* < .01), the path from PEU4 to work-based experimentation was not significant. With both WBL colleagues and experimentation dropped from the analysis, Hypothesis 3 was thus partially supported.

Hypothesis 4a predicted that PEU would be negatively related to psychological distress. The path was significant (-.20, *p* < .01), supporting this hypothesis. Hypothesis 4b predicted that the relationship between PEU and psychological distress is mediated by trust in manager, LMX and workplace learning. The paths from PEU4 to trust in supervisor (.40), LMX (.34), WBL supervisor (.60) and WBL reflection (.25) were significant. However only the path from trust in supervisor was significantly related to psychological distress. The path from PEU to psychological distress was therefore both directly and indirectly significant via trust in supervisor (-.22), supporting Hypothesis 4b. As no significant paths were found between psychological distress and either LMX or workplace learning. Hypothesis 4b was partially supported.

**Fig 1:**
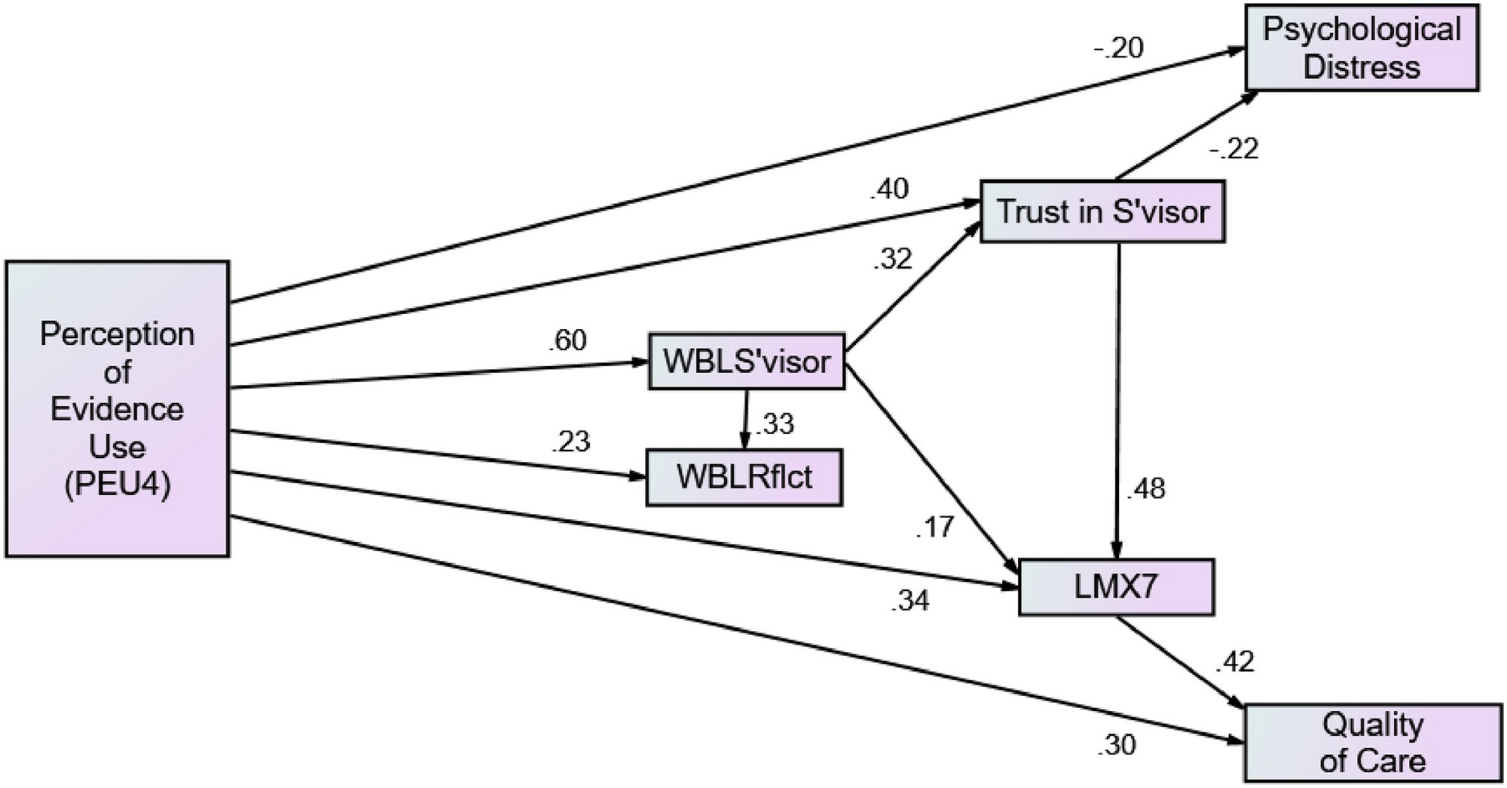
Results of hypothesized model.

Hypothesis 5a predicted that PEU would be positively related to perceptions of quality of care. The path from PEU4 to quality of care was significant (.30, *p* < .01) supporting Hypothesis 5a. Hypothesis 5b predicted that trust in supervisor, LMX, and workplace learning would mediate this relationship. There were no significant paths from WBL supervisor or WBL reflection or trust in supervisor to quality of care. The path from PEU4 to quality of care was mediated by LMX both directly (.42) and indirectly via WBL supervisor (.17) and trust in supervisor (.48). Hypothesis 5b is thus partially supported. Fig 1 displays the final model.

### Tests of alternative models

To confirm that our hypothesized model best represented the data, we estimated several alternative models. We examined Model 0, a path model with all independent and mediated variables as independent correlated variables with direct paths to the dependent variables. The fit statistics shown in Table 9 demonstrate the poor fit of Model 0 compared with the hypothesized model. We then examined other plausible models for alternative explanations. In Model A, PEU4 was positioned as a mediating variable. Model B positioned trust and LMX as mediators with WBL and PEU4 as independent correlated variables. In Model C, the leader variables of PEU4, trust, and LMX were mediated by the WBL variables. Model D positioned PEU4 as the independent variable with WBL, trust, and LMX as mediating variables. Model E included paths from WBL to PEU4, trust, and LMX as mediating variables. Finally, Model F used PEU4 as two factors. None of the models was a better fit than the more parsimonious hypothesized model. These fit statistics are summarized in Table 9.

**Table 9:** Fit statistics for competing models for perceived evidence use (Study 5).

Model	*x* ^2^	Df	*x*^2^/Df	TLI	CFI	RMSEA	SRMR
Hypothesized model	45.09	16	2.82	0.96	0.98	0.08	0.07
0: PEU4, LMX, trust, WBL, no mediation	30.36	5	6.07	0.91	0.97	0.13	0.06
A: LMX, trust, WBL mediated by PEU4	33.74	11	3.07	0.95	0.98	0.08	0.06
B: PEU4, WBL mediated by LMX, trust	33.80	9	3.76	0.94	0.98	0.10	0.06
C: PEU4, LMX, trust mediated by WBL	41.41	14	2.96	0.96	0.98	0.08	0.06
D: PEU4 to uncorrelated WBL, mediated by LMX, trust	262.36	17	15.43	0.68	0.80	0.22	0.12
E: PEU4 from uncorrelated WBL, mediated by LMX, trust	341.07	17	20.06	0.57	0.74	0.25	0.23
F: Alternative two-factor model	124.52	21	5.93	0.90	0.94	0.13	0.08

*Note*. PEU = perceived evidence use; TLI = Tucker Lewis index; CFI = comparative fit index; RMSEA = root mean square error of approximation; SRMR = standardized root mean square residual; LMX = leader–member exchange; WBL = work-based learning.

## Discussion

This study investigated subordinate perceptions of evidence use (PEU) by their managers and its implications for their work-related and organizational experiences. We found that employees are able to discriminate between PEU’s relationships with different types of workplace learning. Notably PEU was strongly associated with learning from the supervisor, and moderately associated with reflective learning. The magnitude of the relationship between PEU and workplace learning from the supervisor was high (.74), suggesting the evidence use by managers may contribute to their subordinates’ ability to learn from those managers. In terms of reflective learning, perceived evidence use may encourage subordinates to make sense of prior experiences in light of newly presented facts and information [76]. Perceiving managerial evidence use can help individuals be strategic about their subsequent actions by encouraging reflection on how best to apply or adapt evidence to new situations. Last, we conclude the PEU4 is sufficient as a research instrument for studying the effects of subordinates’ perceptions managerial evidence use.

Although we cannot determine the specific forms of evidence managers used or the communication processes managers employed to convey their evidence use to subordinates, these first findings suggest organizations that do not encourage evidence-based decision-making are missing out on opportunities to develop their workforce. Our findings suggest that by modeling the evidence use in making decisions, leaders can provide their subordinates with opportunities for workplace learning.

As expected, PEU was substantially related to, yet distinct from both LMX and trust in supervisor. Our findings suggest that leaders may be able to build subordinate confidence in their leadership through careful attention to facts and relevant scientific evidence when making decisions, and through communicating the role that evidence plays in those decisions. Although decisions about what constitutes high-quality evidence are sometimes contested [77], a pattern of evidence-based decision-making can enhance managerial relationships with subordinates.

These results show that managerial evidence use also may enhance employees’ perceptions of the quality of services the organization provides. In the aged care setting, perceptions of quality of care are likely to be an important aspects of organizational performance. Indeed, in other settings, we would expect similar results on other relevant organizational outcomes. In organizations where managers make decisions based on evidence, employees are likely to have increased respect for and commitment to their employer and their managers. In effect, we expect employees to become stronger advocates for their employers when they perceive that organizational decisions are made with attention to evidence. When organizations are seen to offer informal learning opportunities through the communication of decision-making processes, employees are likely to respond with more favorable perceptions of the employer and other indicators of workplace quality. Indeed, “evidence-based management culture” has been described as an important component in a positive learning environment; one that provides a new element in human resource management [4].

We found perception of evidence use to be negatively related to psychological distress. Note, however, that perception of evidence use did not reduce psychological distress via the traditional mechanisms of trust, LMX, and workplace learning that we investigated. Instead, employee perceptions that their managers used evidence directly related to lower psychological distress. It is possible that awareness of one’s manager’s reliance on evidence can reduce employee distress through the psychological ease or confidence evidence use engenders. Alternatively, evidence use may play a role in creating psychological safety in the workplace [78].

### Implications for leadership theory

The relatively large contributions that evidence use made to both LMX and trust in managers offer an opportunity to rethink leadership theory, particularly regarding the nature of effective leadership and behaviors that good leaders manifest. Since the emergence of leadership research in the 20th century, models of leader effectiveness have shifted from focusing on discrete behaviors such as consideration and initiation of structure [79] to assessing different leadership styles, like transformational and charismatic leadership [80]. Evidence use is distinct from these common leadership constructs. A leader employing a transactional style can regularly use evidence related to organizational results to make it clear to subordinates what work they need to get done, while a transformational leader might use findings from research to implement a cutting-edge practice. Conversely, managers characterized by high levels of servant leadership or LMX may have a greater tendency to share the evidence used in their decision-making.

What is particularly important to leadership theory is that organizational environments have become information-rich both in terms of online access to scientific evidence [2] and the array of organizational data [81] available. How leaders make use of information pertaining to their decisions (i.e., evidence) may now itself be an important leadership attribute. In the increasingly complex environment of contemporary organizations, evidence use can aid in addressing the reducible uncertainty inherent in organizational decisions and actions. At the same time, attention to the availability and quality of pertinent evidence can lead to the recognition of how much uncertainty remains (i.e., irreducible uncertainty). Acknowledging what is not known may itself be an important aspect of evidence-based practice, creating more realistic managerial expectations regarding what can be done and what results are to be expected. Observing that one’s manager bases decisions on good quality information may help reduce anxiety associated with the decisions employees are required to execute.

The broader construct of EBM introduces a new dimension to leadership research as well as to practice. As a mode of management practice with implications for organizational outcomes, workplace relationships and learning, evidence-based practice is suggested as an important dimension of leader behavior and a contributing factor in positive leader–member relationships. Its contribution derives from subordinate observations of managerial evidence use in making decisions.

### Future research

Research is needed into the dimensionality of evidence use. Many things can be done with evidence. It can be applied in decision-making, used to evaluate consequences of decisions, shared to explain the rationale for decisions, or reflected upon to promote learning. In the broad practice area of EBM, several forms of evidence play a role, including findings from scientific research, organizational data, practitioner judgment, and stakeholder concerns and perspectives [2]. We recommend further development of evidence assessment measures with particular emphasis on the kinds of evidence used in organizational decisions from science to organizational data, to stakeholder concerns, and personal and professional judgment. Results from our research suggest that evidence use can be a game-changing concept in our understanding of leader behavior, as well as an under-recognized factor in workplace relationships. Drilling down into specific forms of evidence used by managers and in the organization generally could deepen our understanding of important contributors to organizational decisions, learning, and performance.

Perspective is also important in that although we investigated subordinate perceptions of evidence use other pertinent perspectives include those of the individual managers themselves and their superiors. Further research could reveal the extent to which both executive and employee perceptions of managerial evidence use converge with managers’ self-reported evidence use and the implications of the observed degree of convergence. It would be useful to know how those executive and employee observers perceive the quality of the evidence used by managers, and the conditions under which managerial evidence use is salient or ignored. For example, under what conditions might managerial qualities like charisma, authority, and entrepreneurial leadership [82] override the impact of evidence use.

Last, while this study identified possible consequences of perceptions of managerial evidence use, research is needed into its antecedents. Individual ability, motivation, and opportunity to practice all play major roles in engaging in EBP [22]. We know relatively little about specific forms that ability, motivation, and opportunity to practice take in the context of managerial evidence use. Given Ghoshal’s [16] critique of self-interested managerial behavior, research is needed on effects of incentives and supports encouraging managerial evidence use.

### Implications for practice

Managers are likely to create real value for their organizations and benefits for their subordinates by using quality evidence in making decisions. Perceptions of evidence use and sharing reinforce the everyday teaching role managers can perform for their employees. We advise organizations to assess how their managers use evidence by employing the PEU4 scale as part of their larger organizational surveys.

Evidence use by managers in their decisions is part and parcel of building trust-based relationships with subordinates and is likely to foster workplace learning. Managers who run experiments to learn what does and does not work provide employees with a model of openness to feedback and reinforce the value of quality evidence in making decisions and solving workplace problems.

Evidence use is not easy. It can take a special effort to obtain useful scientific and organizational evidence in making organizational decisions. Gathering in-house data requires building supportive infrastructure including information technology capabilities and a willingness to share information. Such investments and efforts can pay off with improved decision quality, organizational learning, and quality relationships with employees. Further, encouraging managers to become more evidence savvy may pose a threat to those managers who fear an emphasis on evidence takes away from their autonomy and personal control. Courage and competence are both required to reduce reliance on intuition and instead make conscientious use of quality evidence.

### Limitations

The cross-sectional design of much of the present research limits our ability to draw inferences regarding causality. Further, the nature of the training and work environment of the caring and nursing staff we studied may limit generalizability of our findings. Although perceptual measures are appropriate when assessing employee perspectives, future research should link employee perceptions with both direct observation and managerial self-reports regarding evidence use. While we considered variables such as trust in supervisor, LMX and workplace learning, we failed to identify potential mediators of the effect of PEU on psychological distress, suggesting more attention is needed into the psychological effects that managerial evidence use has on employees. We suggest psychological safety [82] as a likely underlying factor, but more theory and research is needed regarding PEU’s effect on reducing employee distress. We note the RMSEA values associated with smaller degrees of freedom and have provided alternative fit measures [83]. Finally, despite Studies 2, 3 and 4 having verified the reliability and validity of the new measurement on separate datasets, additional field research using the PEU measure is needed to confirm and extend our findings to identify potential overlaps with other constructs and boundary conditions. Future research should consider whether there are differences in perceptions of evidence use depending on whether an individual or the broader organisation is the ultimate target, and whether those differences have parallel effects on individual or organizational dependent variables.

## Conclusion

This research demonstrates specific effects of perceptions of managerial evidence use. Using an assessment with robust psychometric properties, these findings advance our understanding of EBM by identifying ways that PEU by managers can enhance the employee workplace experience. These results highlight the potential game-changing role that managerial evidence use can play in relationships between leaders and their subordinates. In information-rich environments, how contemporary leaders make use of evidence can be a telling leadership quality itself.
